# Revisiting the public health implications of the United States–Mexico– Canada agreement

**DOI:** 10.1186/s12992-020-00579-y

**Published:** 2020-06-05

**Authors:** Tolulope Anthony Adekola

**Affiliations:** School of Law, City University of Hong Kong, Hong Kong, Hong Kong SAR

**Keywords:** Public health, USMCA, Pharmaceutical patent, Biologics

## Abstract

This commentary re-examines a recent article by Labonté et al on the recent changes to two relevant provisions relating to patent rights in the final version of the United-States-Mexico-Canada Agreement (USMCA). Although the USMCA’s final revised version does not add more pharmaceutical patent protection than those that already exist in the three trading partners, the agreement has done little to enhance access to generic medicines and biosimilars.

Labonté et al. have called our attention to the persistent imbalance in the global intellectual property system as evidenced in the TRIPS-plus^1^ provisions incorporated into the newly signed United States Mexico Canada Agreement (USMCA) [1]. The authors carried out an extensive analysis of specific provisions in the USMCA which have far-reaching implications on public health and most importantly, access to generic medicines and biosimilars [1].

Labonté and his colleagues found that rather than enhance public health, the USMCA is capable of increasing the price of drugs in Canada and Mexico [1]. However, some of the critical provisions that formed the crux of their analysis ended up being removed from the final version of the USMCA in December 2019 [2]. While the authors should be pleased that some of the controversial provisions are no longer part of the agreement’s final version, it, however, makes some of their arguments overtaken by events.

On 10 December 2019, Canada, Mexico and the United States agreed after further negotiations to amend the earlier draft of the USMCA. The latest and final version of the agreement removes two basic provisions relating to patent rights [2]. The first relates to the obligation earlier imposed on parties to provide patents for ‘new uses, new methods and new processes’. Labonté et al. had relied on this earlier provision (Article 20:36 para 2) to argue that the USMCA encourages ‘evergreening’ of pharmaceutical patents. They raised concerns that the provision may have the effect of promoting patents for minor improvements while delaying the entrant of generic manufacturers [3]. As it stands, the requirement has now been explicitly deleted.

Secondly, the 10-year protection earlier accorded to biologic drugs has also been deleted [4]. Labonté and his colleagues had argued that the 10-year period of ‘effective market protection’ for biologics was the longest ever in any trade agreement. They analyzed the foreseeable implication of the provision on the need to guarantee the kind of market competition needed to drive affordable biosimilars. Indeed, had the provision not been deleted, Canada, which currently accords eight-years market protection for biologics would have been compelled to extend the protection upwards to 10 years [5]. On the other hand, Mexico seems to be the biggest beneficiary of the removal of the biologic drugs protection requirement. Currently, Mexico does not offer data protection for biologic drugs; as such, the earlier 10-year data protection requirement imposed by the USMCA would have come at a high cost. Now that the final version of the USMCA no longer imposes an obligation on parties to provide test data protection to biologic drugs, the concerns expressed by Labonté and his colleagues have been cleared.

Despite the removal of the two provisions relating to ‘evergreening’ and protection of biologics, the findings of Labonté and his colleagues as touching the public health and regulatory implications of the retained provisions in the USMCA are very valid and relevant. Even though the final version of the USMCA does not add more intellectual property protections than those that already exist in these countries, the agreement has done little to enhance public health [6].. The final version of the USMCA has now been ratified by the three trading partners and is scheduled to come into effect on July 1, 2020. From whichever angle one appraises the effect of the agreement’s revision, the trade deal still primarily benefits the United State’s corporate and political interests. However, both Canada and Mexico were, to some extent, able to protect some of their interests. As argued by Labonté and his colleagues, the ratcheting up of intellectual property rules is indeed a symptom of a more extensive disorder, which reveals the deteriorating imbalance in the global intellectual property regime [7]. In the United States, for instance, despite the legislative proposal to the congress to spool back the current 12-year market exclusivity for biologic drugs to 7 years, nothing has changed. The USMCA’s earlier provision on biologics, which could have further reduced the protection by 12 years in the United States, has been removed. The USMCA, rather than ameliorate the existing effect of intellectual property protection on access to generic medicines and biosimilars in the region, left the situation the same way it is.

Laborté and his colleagues’ treatment of intellectual property rights and regulatory constraints in the USMCA indeed offers new perspectives that enrich an already active field of study. We are thankful to them.

## Data Availability

Data sharing is not applicable to this article as no datasets were generated or analyzed during the current study.

## Footnotes

1 TRIPS-Plus refer to the extension of Intellectual property protection standards beyond the minimum requirements set by WTO’s Agreement on Trade-Related Aspects of Intellectual Property Rights (TRIPS).
